# I-131 mIBG Scintigraphy Curie Versus SIOPEN Scoring: Prognostic Value in Stage 4 Neuroblastoma

**DOI:** 10.4274/mirt.52533

**Published:** 2018-10-09

**Authors:** Saima Riaz, Humayun Bashir, Saadiya Javed Khan, Abid Qazi

**Affiliations:** 1 Shaukat Khanum Memorial Cancer Hospital and Research Centre, Clinic of Nuclear Medicine, Lahore, Pakistan; 2 Shaukat Khanum Memorial Cancer Hospital and Research Centre, Clinic of Pediatric Oncology, Lahore, Pakistan; 3 Canal Bank, Clinic of Surgery, Lahore, Pakistan

**Keywords:** I-131 mIBG scan, neuroblastoma, Curie scores, SIOPEN scores

## Abstract

**Objective:**

I-131 mIBG scan semi-quantitative analysis with modified Curie and the International Society of Pediatric Oncology Europe Neuroblastoma (SIOPEN) scoring systems is helpful in the evaluation of disease extent and has prognostic impact in stage 4 neuroblastoma.

**Methods:**

Retrospective, cross-sectional analysis of baseline I-131 mIBG scans in 21 patients with stage 4 or 4S neuroblastoma diagnosed between January 2007 and December 2015. All scans were assessed for Curie and SIOPEN scores. Distribution of scores was evaluated for risk factors i.e. age at diagnosis (>18 months) and early relapse (within 12 months). A curie score <2 and SIOPEN score <4 at diagnosis were correlated with event-free survival (EFS) and overall survival (OS).

**Results:**

The data set comprised of 12 (57%) males and 9 (43%) females. Patients with age >18 months (n=9) at diagnosis or early relapse (n=9) had higher Curie [mean 5+7.5 standard deviation (SD), p=0.004] and SIOPEN (mean 5.2+10.8 SD, p=0.02) scores. Patients with a Curie score <2 and a SIOPEN score of <4 had better EFS and OS than patients with higher scores. Curie: 5-year EFS=Curie <2 (79%) versus Curie >2 (33%) (p=0.03); 5-year OS=Curie <2 (56%) versus Curie >2 (36%) (p=0.01). SIOPEN: 5-year EFS=SIOPEN <4 (70%) versus SIOPEN >4 (17%) (p=0.002); 5-year OS=SIOPEN <4 (58%) versus SIOPEN >4 (17%) (p=0.04). There was no statistically significant difference between the two scoring systems in terms of survival predictive value (Hazard ratio 2.38, 95% CI: 0.33-16.9, p=0.38).

**Conclusion:**

I-131 mIBG Curie and SIOPEN scores have prognostication value in stage 4 neuroblastoma and should be routinely applied. Higher scores predict unfavorable prognosis.

## Abstract (tr)

**Amaç:**

I-131 mIBG sintigrafinin modifiye Curie ve International Society of Pediatric Oncology Europe Neuroblastoma (SIOPEN) skorlama sistemleri ile yarı-kantitatif analizi evre 4 nöroblastomda hastalığın yayılımını değerlendirmede yararlıdır ve prognostik etkisi bulunmaktadır.

**Yöntem:**

Ocak 2007 ile Aralık 2015 tarihleri arasında, evre 4 veya 4S nöroblastom tanısıyla I-131 mIBG sintigrafi yapılmış 21 hastanın retrospektif, kesitsel analizi yapıldı. Tüm tetkikler Curie ve SIOPEN skoru ile değerlendirildi. Tanı yaşı (>18 ay) ve erken nüks (12 ay içinde) gibi risk faktörlerine göre skor dağılımı değerlendirildi. Tanı anında Curie skoru <2 ve SIOPEN skoru <4 hastalıksız (HGS) ve genel sağkalım (GS) ile ilişkili idi.

**Bulgular:**

Çalışmaya 12 (57%) erkek ve 9 (43%) kadın dahil edildi. Tanı anında yaşı >18 ay olan hastalar (n=9) veya erken nüks edenlerde (n=9) Curie [ortalama 5+7,5 standart deviasyon (SD), p=0,004] ve SIOPEN (ortalama 5,2+10,8 SD, p=0,02) skorları daha yüksek bulundu. Curie skoru <2 ve SIOPEN skoru <4 olan hastaların HGS ve GS daha yüksek skorlu hastalardan daha iyiydi. Curie: 5-yıl HGS=Curie <2 (%79) vs Curie >2 (%33) (p=0,03); 5-yıl GS=Curie <2 (%56) vs Curie >2 (%36) (p=0,01). SIOPEN: 5-yıl HGS=SIOPEN<4 (%70) vs SIOPEN >4 (%17) (p=0,002); 5-yıl GS= SIOPEN <4 (%58) vs SIOPEN >4 (%17) (p=0,04). Her iki skorlama sistemi arasında sağkalım öngörücü değer açısından istatistik olarak anlamlı farklılık saptanmadı (Risk oranı: 2,38, %95 CI: 0,33-16,9, p=0,38).

**Sonuç:**

I-131 mIBG Curie ve SIOPEN skorlarının evre 4 nöroblastomda prognostik öngörü değeri vardır ve rutin olarak kullanılmalıdır. Yüksek skorlar daha kötü prognozu göstermektedir.

## Introduction

Neuroblastoma is the most common extra-cranial solid malignant tumor in children. It originates from the sympathetic nervous system, most frequently from the adrenal medulla. The heterogeneous clinical behavior of neuroblastoma is dependent on numerous clinical as well as biological features (1). Neuroblastoma is diagnosed over a wide age range, from birth through young adulthood. Older age at diagnosis indicates a grim survival. Poor prognostic factors include: Age >18 months, NMYC amplification, poorly differentiated, advanced stage disease, and indistinguishable tumor histology (2). Approximately 70% of neuroblastoma patients present with metastatic disease (3). With the current treatment approaches, age at diagnosis has proven to be one of the most influential predictors of the outcome. In patients older than 1 year, the 5-year disease-free survival rates for stage 4 neuroblastoma have been reported to be 30-46% (4).

Treatment regimens including induction therapy, autologous stem cell transplantation, better radiotherapy techniques and immunotherapy have led to improvements in disease-free survival. Tailoring treatment to risk group stratification has improved outcome. The capacity to identify both biologic and clinical prognostic markers of response has the benefit of selecting treatment therapies (5).

Radioiodine labeled metaiodobenzylguanidine (mIBG) is an aralkylguanidine, structurally parallel to norepinephrine. Almost 90% of neuroblastoma concentrate mIBG within the marrow, soft-tissue sites of disease or cortical bone (6,7). Since I-123 labeled mIBG is not available in our part of the world, I-131 mIBG is used.

## Materials and Methods

Retrospective data of baseline I-131 mIBG scans was analyzed in patients with stage 4 or 4S neuroblastoma registered between January 2007 and December 2015. Our data set included 21 patients, aged 0.5 to 12 years. Twelve were males (57%) and 9 (43%) females.

This retrospective study has been approved by the Institutional Review Board of Shaukat Khanum Cancer Hospital and Research Centre, Lahore.

### Imaging

Radiotracer dose in the range of 37 to 55 MBq I-131 MIBG was injected intravenously adjusted to patients’ weight according to EANM recommendations. Scanning was acquired at 24 and 48 hours using Siemens Symbia T16 camera, 128x128 matrix for planar scan, bed movement 7-8 cm/min.

### Semi-quantitative Analyses

Semi-quantitative evaluation of all scans was performed using the Curie and International Society of Pediatric Oncology Europe Neuroblastoma (SIOPEN) scoring methodology.

Initially developed in 1995, scoring is based on the presence of mIBG uptake in multiple anatomic regions (8,9). Ten different sites were scored, including 9 skeletal sites (head, chest, T-spine, L-spine, pelvis, upper arms, lower arms, femurs, and lower legs) and an additional tenth site for soft-tissue lesions.

The International Neuroblastoma Risk Group (INRG) Staging System (INRGSS) is an imaging defined staging and risk assessment system. According to the SIOPEN semi-quantitative scoring method, the skeleton was divided into 12 anatomical body segments as follows: the skull, the thoracic cage, the proximal right upper limb, the distal right upper limb, the proximal left upper limb, the distal left upper limb, the spine, the pelvis, the proximal right lower limb, the distal right lower limb, the proximal left lower limb and the distal left lower limb. The extent and pattern of skeletal mIBG involvement was scored using a 0-6 scale to discriminate between focal discrete lesions and patterns of more diffuse infiltration (10).

Distribution of scores was evaluated for two risk factors i.e. age at diagnosis (>18 months) and early relapse (within 12 months).

### Statistical Analysis

The data were analyzed by the Kaplan-Meier method using IBM SPSS statistics 20 program. A Curie score <2 and SIOPEN score <4 (est defined cut-off) at diagnosis were correlated with event-free survival (EFS) and overall survival (OS) (11). Log-rank test with a p value of less than 0.05 was used to evaluate the differences between groups. Cox proportional hazards regression model applying the enter method was used to estimate hazards ratio (HR) for analysis.

## Results

The data set included 12 (57%) males and 9 (43%) females (age range: 4 months to 12 years). All had stage 4 disease in terms of osteomedullary metastases (n=9), soft issue metastases (n=8) and cytological bone marrow involvement (n=4) (Table 1).

Out of total 21 patients, 12 (57%) were younger than 18 months, while 9 (43%) older than 18 months of age. Nine patients (57%) had either relapse within 12 months after diagnosis or primary progressive disease.

### Semi-quantitative Scoring

Overall Curie score ranged from minimum 1 to a maximum score of 27 (average 5.9±7.9 S). SIOPEN scores ranged between 0 to a maximum score of 48 [average 6.1±11.6 standard deviation (SD)].

On Curie scores analysis, 10 patients had <2 and 11 had >2 Curie scores. SIOPEN score <4 was calculated in 15 and >4 in 6 patients.

### Distribution of mIBG Score by Risk Factors

In patients with age <18 months at diagnosis, Curie scores were 2.7±4.2 SD and SIOPEN scores were 1.2±3.2 SD. For age >18 months, Curie scores were identified as 10.4±9.7 SD and SIOPEN scores 12.8±15.4 SD. On bivariate analysis, the scores were higher in age >18 months (p=0.002 for Curie and p=0.018 for SIOPEN scores).

**Figure 1:**
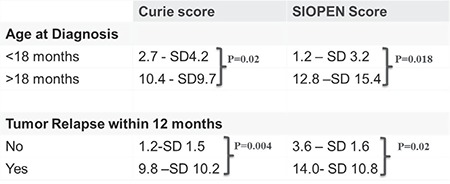
Distribution of Curie and International Society of Pediatric Oncology Europe Neuroblastoma scores with reference to risk factors *SD: Standard deviation*

In reference to relapse, patients with early relapse had Curies scores 9.8±10.2 SD, and SIOPEN scores were 14.0±10.8 SD. In comparison to this, patients who did not show early relapse, the Curie scores were 1.2±1.5 SD, while SIOPEN scores were 3.6±1.6 SD. The scores tended to be higher in patients with early relapse within 12 months in comparison to patients who did not show early relapse (p=0.004 for Curie and p=0.02 for SIOPEN scores). The details of these scores have been displayed in Figure 1.

### Disease Outcome and Survival Analysis

Out of 10 patients with Curie score <2, 2 (20%) relapsed and 2 (20%) died, while with Curie score >2, 7 out of 11 (63.6%) relapsed and 6 (54.5%) died.

On analysis of SIOPEN scores, out of 15 patients with score <4, 4 (27%) relapsed and 4 (27%) died, while with scores >4, 5 out of 6 (83%) relapsed and 4 (67%) died.

Comparative analyses demonstrated that Curie score <2 and a SIOPEN score of <4, respectively, had better EFS and OS than patients with higher scores. 5-years EFS for Curie score <2 was 79% versus 33% for score >2 (p=0.03). 5-year OS was 56% for Curie score <2 versus 36% for score >2 (p=0.01).

**Figure 2:**
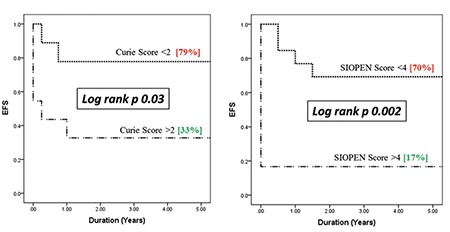
Curie and International Society of Pediatric Oncology Europe Neuroblastoma scores at diagnosis and event-free survival SIOPEN: International Society of Pediatric Oncology Europe Neuroblastoma, *EFS: Event-free survival*

**Figure 3:**
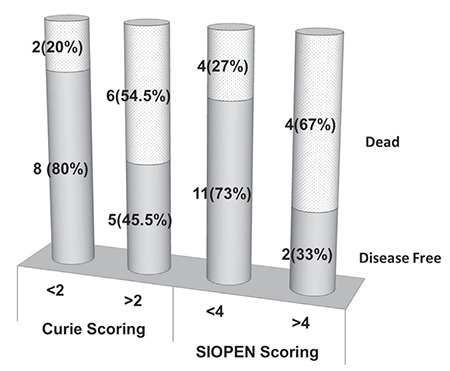
Curie and International Society of Pediatric Oncology Europe Neuroblastoma scores at diagnosis and overall survival *SIOPEN: International Society of Pediatric Oncology Europe Neuroblastoma*

Based on SIOPEN scoring, 5-year EFS was found to be 70% for score <4 as compared to 17% for score >4 (p=0.002). Similarly, 5-year OS for score <5 was 58% versus 17% for score >4 (p=0.04). The Kaplan-Meier disease-free survival distributions based on these low or high Curie and SIPOEN scores were significantly different (Figure 2, 3).

### Comparing Curie and SIOPEN Scoring Systems in Predicting Prognosis

On bivariate analysis, there was no statistically significant difference between the two scoring systems in terms of survival predictive value [HR: 2.38, 95% confidence interval (CI): 0.33-16.9, p=0.38].

## Discussion

Accurate disease staging is essential for an optimized treatment plan. The outcome is related to disease extent in stage 4 neuroblastoma. Radioiodine labeled mIBG semi -quantitative scoring systems are used to estimate diasease burden. We selected two poor prognostic factors, age older than 18 months and early relapse, to evaluate the validity of mIBG scores (12).

In most of the earlier studies, age <12 months has been taken as the poor prognostic indicator. However, as shown by Moroz et al. (12,13), 18 months is a powerful indicat or of unfavorable outcome as the cut off for age-of-diagnosis. Likewise, time to first relapse influences survival. London et al. (13), demonstrated that mortality risk is higher in patients who relapse early (14).

Scores tended to be higher in patients with age >18 months at diagnosis and early relapse. We found a statistically significant positive correlation between higher scores and poor prognostic factors. In this regard, radioiodine mIBG scan at baseline with high scores can be taken as an indicator of poor outcome. Various studies have shown different cut-off values for mIBG score systems (14,15), and there are several previous studies where no significant prognostic impact of the initial mIBG score was reported (16,17).

However, Decarolis et al. (11), reported the best cut-off for the Curie score as 2 and that for the SIOPEN score as 4, which significantly discriminated between poor and more favorable outcomes. We evaluated the validity of these cut-off scores in our referral group. Patients who tended to have higher scores at baseline had higher frequencies of disease relapse and deaths. Outcome was significantly better in lower scores with better EFS and OS when compared with higher scores.

An INRG task force led by Matthay et al. (10) has examined both methodologies as a potential prognostic marker for outcome determination. The SIOPEN scoring methodology is currently being used in SIOPEN high risk neuroblastoma trials, with Curie scoring used in COG trials.

In a prior review of COG-A3973, COG investigators were unable to identify a mIBG (Curie) score at diagnosis that correlated with outcome (14). Ladenstein et al. (15) studied the baseline scores at diagnosis and reported SIOPEN mIBG score to be highly prognostic of outcome in two independent data sets, SIOPEN/HR-NBL1 and COG-A3973. In comparison, our data set comply with findings of Decarolis et al. (11). We found that the prognostic value of Curie and SIOPEN scores were similar. Although SIOPEN scores do not include soft tissue disease, both scoring systems were found to have similar prognostic significance with no statistically significant difference (HR: 2.38, 95% CI: 0.33-16.9, p=0.38).

### Study Limitation

The limitation of this study is the limited number of follow-up I-131 mIBG scans, to provide statistically significant outcome analyses based on post-induction Curie and SIOPEN scores. Our data needs further extension with future prospect to report the validity of these scores in therapeutic response evaluation and to develop mIBG scoring systems based response criteria.

## Conclusion

In conclusion, our study demonstrates the feasibility of mIBG scoring systems, which have prognostication value in stage 4 neuroblastoma. These scores are not used in routine clinical practice. However, with advancement in therapeutic options in stage 4 neuroblastoma, the implementation of mIBG scoring systems can be helpful in more precise prognostication based treatment.

## Figures and Tables

**Table 1:**
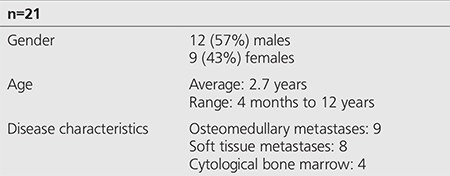
Patient and disease characteristics
